# Scaffolds and Extracellular Vesicles as a Promising Approach for Cardiac Regeneration after Myocardial Infarction

**DOI:** 10.3390/pharmaceutics12121195

**Published:** 2020-12-09

**Authors:** Melody Riaud, M. Carmen Martinez, Claudia N. Montero-Menei

**Affiliations:** 1 SOPAM, U1063, INSERM, UNIV Angers, SFR ICAT, F-49800 Angers, France; melody.riaud@univ-angers.fr; 2 CRCINA, UMR 1232, INSERM, Université de Nantes, Université d’Angers, F-49933 Angers, France

**Keywords:** regenerative medicine, myocardial infarction scaffolds, extracellular vesicles

## Abstract

Clinical studies have demonstrated the regenerative potential of stem cells for cardiac repair over the past decades, but their widespread use is limited by the poor tissue integration and survival obtained. Natural or synthetic hydrogels or microcarriers, used as cell carriers, contribute to resolving, in part, the problems encountered by providing mechanical support for the cells allowing cell retention, survival and tissue integration. Moreover, hydrogels alone also possess mechanical protective properties for the ischemic heart. The combined effect of growth factors with cells and an appropriate scaffold allow a therapeutic effect on myocardial repair. Despite this, the effects obtained with cell therapy remain limited and seem to be equivalent to the effects obtained with extracellular vesicles, key actors in intercellular communication. Extracellular vesicles have cardioprotective effects which, when combined proangiogenic properties with antiapoptotic and anti-inflammatory actions, make it possible to act on all the damages caused by ischemia. The evolution of biomaterial engineering allows us to envisage their association with new major players in cardiac therapy, extracellular vesicles, in order to limit undesirable effects and to envisage a transfer to the clinic. This new therapeutic approach could be associated with the release of growth factors to potentialized the beneficial effect obtained.

## 1. Myocardial Infarction

Coronary heart disease, more specifically myocardial infarction (MI), is the leading cause of death in the world according to the World Health Organization, representing about 31% of global mortality, and in particular, MI accounts for 13% of mortality [1]. The high incidence of this disease in developed countries and constant aging of the population make it one of the primary areas of research aimed at restoring cardiac function after MI. Ischemia results from the obstruction of an artery that supplies the heart with blood and oxygen due mainly to partial or total detachment of atheroma plaques, leading to disruption or even cessation of the function of the heart [2]. This phenomenon can occur in a sudden or delayed manner; for the latter, the establishment of a secondary vascular network leads to the replacement of the preexisting damaged vascular network and limits the damage observed as a result of hypoxia.

**Figure 1:**
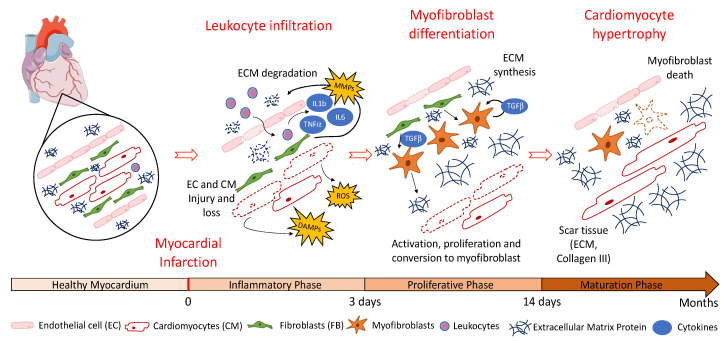
Different cellular actors are involved in the inflammatory, proliferative and maturation phases following myocardial infarction (MI).

Three phases are described following the occurrence of an MI resulting in left ventricular (LV) remodeling: the inflammatory phase (0–3 days), followed by the proliferative phase (3–14 days) and finally the maturation phase (14 days to 2 months) [3] (Figure 1). During the inflammatory phase, initiated by necrosis and apoptosis induced by ischemia, with or without reperfusion, free radicals and chemokines including reactive oxygen species (ROS), damage-associated molecular patterns (DAMPs), tumor necrosis factor-α (TNF-α), interleukin 1β (IL1β) and interleukin 10 (IL10) are released [4]. This gradient of chemokines as well as the action of matrix metalloproteinases (MMPs) secreted by fibroblasts and allowing the degradation of the extracellular matrix (ECM) lead to a massive infiltration of leukocytes and the expression of pro-inflammatory cytokines that recruit pro-inflammatory fibroblasts [5]. This recruitment allows the establishment of the proliferative phase, in which the major players are cardiac fibroblasts. Their differentiation towards a myofibroblast phenotype induces a significant secretion of collagen III, participating in the establishment of scar tissue to replace the cardiomyocytes lost during MI. Most often, the subsequent remodeling of the LV wall rather than cardiomyocyte loss during MI is the main cause of the heart failure. The persistence of myofibroblasts within the myocardium and, more specifically, the secretion of tumor growth factor-β (TGF-β), contribute to their survival and, thus, to the establishment of non-functional cardiac fibrous tissue [6].

Finally, during the maturation phase, scarring is effective and stiffening causes diastolic dysfunction, contraction problems and cardiac arrhythmias [7,8]. These alterations are also caused by cardiomyocyte hypertrophy in order to compensate for the deleterious effects elicited by LV remodeling. This leads to issues such as heart failure and heart rhythm disorders that irreparably impact life quality for patients. The time and delay to apply the treatment, following MI, determines the severity of the resulting ischemic lesions, particularly on the surface of the myocardium. The severity of lesions is also correlated with the death of hypoxic myocardial cells that engage in apoptotic and necrotic processes within the first few hours after coronary occlusion [6,9]. Indeed, once cardiomyocyte death occurs and scar tissues, composed mainly of collagen and ECM proteins provided by fibroblasts, are set in place, the heart loses part of its cardiac functionality due to remodeling of the ventricle [10].

During MI, immediate therapeutic interventions are both curative and preventive: curative by the installation of mechanical supports to correct the arterial abnormality caused by a lack of oxygen and nutriments induced by ischemia and preventive by vasodilator medication consisting of anti-aggregants to reduce the risk of blood clot formation and to prevent atheromatous risk factors. Although heart transplantation remains the most effective treatment, it is only offered to the youngest patients with the best chances of survival after transplantation, given the low availability of grafts and despite the burdensome life-long immunosuppressive treatment. Regenerative medicine, particularly cell therapy, has emerged as a promising interventional strategy. Indeed, current therapies are designed to promote improvement of endothelial function, including neovascularization, and inhibition of cardiomyocyte death to preserve cardiomyocyte contractility. Thus, many clinical trials have been conducted in recent decades, all with the objectives of neovascularization and/or cardiomyogenesis in order to obtain tissue regeneration (Table 1). In these trials, many cells have been used with multiple but different objectives for each one [11]. Despite strong preclinical data, the results obtained with clinical trials vary depending on the cells used and do not always show the same benefits.

### Clinical Trials Based on Cell Therapy for Cardiac Regeneration

Skeletal myoblasts were the first cells used for cardiac regeneration studies because of their abundance and contractile properties, which should help to recover lost contractility caused by MI. However, their transplantation induces an increase in arrhythmia without improvement in cardiac function [12,13,14]. In addition, the study published by Pagani et al. [15] highlights the high mortality of cells following their injection, notwithstanding their ability to form viable contractile tissue. An alternative strategy is based on mesenchymal stromal cells (MSCs) derived from bone marrow (BM) because of their ease of isolation and their capacity to secrete tissue repair factors promoting cardiac regeneration. Moreover, these cells may engage into an endothelial- or cardiomyocyte-like phenotype in vivo [16]. Many clinical trials were performed with BM-derived stem cells showing the feasibility of these studies without side effects caused by the presence of these cells [17,18,19]. However, most of the clinical trials using autologous BM stem cells, including MSCs or hematopoietic stem cells (HSCs), show uncertain results with regard to the benefit provided by these cells, and no clear improvement in cardiac function could be demonstrated [20,21,22,23]. Moreover, these cells appear to be unable to differentiate in vivo into functional cardiomyocytes. These studies underscore the necessity to better control the major study parameters, such as the size of the cohort, the cell quantity to be used, the time of injection of the treatment as well as the route of administration [24]. The choice of cell type is also crucial, and the isolation method needs to be standardized to limit the strong cell heterogeneity obtained [21,22,23]. To overcome this heterogeneity, the BAMI clinical trial was set up with the aim of standardizing the methods of bone marrow mononuclear cell (BMMC) collection, manipulation and administration [25]. This study, which was completed just a few months ago, is expected to determine the effect of BM stem cells on cardiac function.

Another approach is to implant cardiac progenitors to replace damaged cardiomyocytes in order to enhance cardiac regeneration. In fact, it has been shown that naive or activated cardiac progenitors implanted at the site of MI allow the secretion of endogeneous growth factors (GF) promoting cardiac repair [26]. Other cells also have interesting properties in cardiac regeneration. Indeed, cardiac stem cells (CSCs) display a lack of recognition by the immune system due to the lack of expression of major histocompatibility complex class II antigens and B7 costimulatory molecules, preventing their recognition by lymphocytes [27]. They also have the potential to differentiate into smooth muscle cells, cardiomyocytes or endothelial cells, which would allow them to replace the lost cells and, in this way, concur to cardiac regeneration. However, the benefit of the use of CSCs remains unclear since a preclinical study has shown an improvement in the LV ejection fraction [28] whereas, conversely, the CADUCEUS clinical trial is unable to demonstrate an increase in this same parameter [29].

The use of induced pluripotent stem cells (iPSCs) is also attractive due to their pluripotent potential, suggesting that they have the ability to differentiate into cardiomyocytes or endothelial cells. Recently, reprogrammed iPSCs engaged in differentiation into mature cardiomyocytes has been envisioned and are currently being evaluated for MI cell therapy in preclinical studies [30]. However, the use of iPSCs in the clinic still remains controversial due to their genetic reprogramming, which can cause a risk of tumor transformation [31].

Overall, all these studies generally lead to the same conclusion regarding the obstacles encountered, which are problems of survival, differentiation and long-term integration of cells within the hostile ischemic microenvironment, furthermore presenting immune cells secreting pro-inflammatory cytokines. These transplanted cells can only fully exert their action in the presence of a favorable microenvironment, composed of GF, or with an ECM stimulating the survival, differentiation and correct integration into the host tissue [32]. In this regard, combined studies of cells associated with cell carriers or scaffolds providing that favorable microenvironment are being performed. Indeed, the first clinical trial to treat heart failure conducted with embryonic stem cells (ESCs) in human patients (ESCORT) was performed with cells administered as a cardiac patch composed of a fibrin-based hydrogel containing the cells [33,34]. The fibrin patch provided mechanical support through its elasticity properties but also exerted a complementary effect by providing a matrix to optimize the early retention and survival of the transplanted ESCs. Nevertheless, ethical issues raised by the acquisition and use of ESCs represent significant barriers to their widespread use [35].

In 2020, more than 700 clinical trials with cardiovascular cell therapy as the first line are being conducted, but only few phase III trials are ongoing, suggesting a limited use of cell therapy products in the clinic in the near future. Some clinical trials have demonstrated a real therapeutic effect. The BOOST trial marked an improvement in left ventricular ejection fraction after 6 months [19] but not after 18 months [36]. In an equivalent way, the REPAIR-AMI clinical trial showed a significant improvement in myocardial performance after 4 months [37]. It can be noted that most clinical trials showed very distinct and contradictory results with the use of stem cells, such as ASTAMI and REPAIR-AMI, which might at least partly be explained by differential cell processing used in these studies. Nevertheless, ongoing research in this field has recently shown that the use of synthetic miRNAs incorporated into the cells prior to transplantation can also ameliorate the efficacy of cell therapy. This aspect will not be covered in this review, but more information can be found in other reviews [38,39,40]. The encouraging findings of the ESCORT clinical trial, which are also supported by in vivo studies in murine models using cell carriers, demonstrate the importance and utility that scaffolds can have in tissue regeneration and will be developed further.

## 2. Scaffolds for Cardiac Applications

As described above, in cell therapy studies, the cells encounter survival and integration problems mainly due to the microenvironment in which they are transplanted. Indeed, the infiltration of leukocytes and macrophages following ischemia leads to the secretion of soluble factors, cytokines and chemokines such as TNF-α, IL-1 or IL-6, known to be involved in inflammatory phenomena. As a result, hypoxic and inflammatory conditions make the microenvironment unfavorable to cell survival and differentiation. In order to overcome these cell issues, cell carriers have been developed to favor cell survival and integration despite the harmful environment. The strategy is to design and use cell carriers with structural and biological characteristics based on criteria specific to the cell to be used and the application envisioned. In the context of cardiac regeneration, the choice of a scaffold is subject to significant constraints due to the contractile properties of the organ. To satisfy these constraints, hydrogels displaying biomechanical and elastic properties are the most used since they can also participate in restoring part of the lost cardiac function [41,42]. In order to enable cell engraftment, it has also been shown that the scaffold as well as the ECM deposition on scaffolds may allow cell anchorage essential for cell survival and growth [43].

There are a wide variety of scaffolds produced from different natural or synthetic biomaterials. Natural scaffolds allow biological stimuli induction but require delicate processes of reproducibility and purification. On the contrary, synthetic scaffolds, for which manufacturing processes are mastered, need to generally be functionalized to provide the desired biological signals to the endogenous and/or grafted cells. These two types of scaffolds, although different in origin, remain complementary in the cardiac regeneration field and are both used. Other important parameters to be considered when using a scaffold for tissue regeneration include their biodegradability and biocompatibility. If the scaffold used is biodegradable, the physical support that it provides remains until its complete degradation, allowing cell migration and integration within the parenchyma without the need to surgically remove them. In addition, it is necessary that they are not cytotoxic but biocompatible in order to obtain a beneficial effect with no or minimal immunological reaction [43,44,45]. A local route of delivery of these biodegradable scaffolds is also important to limit systemic side effects. Moreover, the mode of administration needs to be easy and fast due to constant contraction of the myocardium.

**Figure 2:**
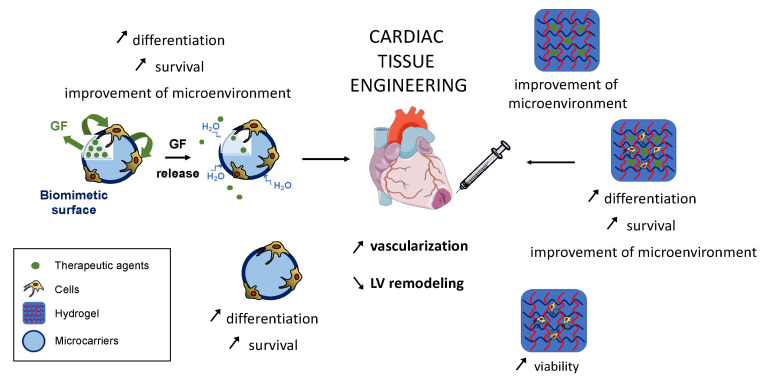
Natural and synthetic scaffolds used in cardiac tissue engineering.

Injectable scaffolds, that can be rapidly and directly injected with minimal tissue invasion, particularly if small in size, can be used as cell carriers and as drug delivery vectors. On the other hand, this drug delivery application may reduce the cost of treatment by a prolonged action of the therapeutic strategy treatment. Many other criteria, such as the surface characteristics of the scaffold, which are described more extensively in the literature [43,46,47], need also to be considered when designing a scaffold for cardiac applications. For the reasons stated above, this review will focus on the use of two types of injectable and biodegradable scaffolds in cardiac ischemia approved by the Food and Drug Administration (FDA): hydrogels, from natural biomaterials, most commonly used in cardiac tissue engineering, and microcarriers, formulated from synthetic polymers providing 3-dimensional anchorage support for transplanted cells. As both scaffolds are able to convey cells and/or therapeutic molecules, these aspects will also be developed in this review (Figure 2).

### 2.1. Biomaterials Used as Therapeutic Scaffolds

Among the scaffolds considered for cardiac regeneration, the vast majority are hydrogels due to their rheological properties that respond to the pressure and contraction forces exerted by the heart muscle. There is a wide choice of biomaterials that can be adapted as closely as possible to the therapeutic usefulness. Usually, many natural injectable hydrogels have been tested in cardiac tissue [48,49] such as fibrin and alginate [50], collagen [51], matrigel [52] or chitosan [53]-based hydrogels. In humans, a fibrin hydrogel injected into a patient’s myocardial scar can restore contractility in a previously akinetic region of the heart and can preserve cardiac function after MI [54]. It was also shown that a single injection of a hydrogel, obtained from a solution based on decellularized pig hearts in which only the ECM remains, promotes endogenous cell multiplication and does not cause cardiac arrhythmias [55,56]. This hydrogel also preserves post-MI cardiac functions by allowing the recruitment of Th2 lymphocytes, known for their production of anti-inflammatory cytokines as IL-4, IL-10 or IL-13, involved in the inhibition of LV remodeling by limiting the strong inflammation response induced by MI. Beneficial effects have been also described with the use of natural collagen-hydrogel. Indeed, collagen is abundantly distributed in the ECM with a very high biocompatibility and biodegradability [57]; however, its low elastic modulus limits its mechanical integration and stabilization [51]. Chitosan-based hydrogels are also widely used due to their intrinsic antibacterial properties, biodegradability and the production of minimal immune responses in humans with no chronic inflammatory response [58]. Indeed, chitosan is a positively charged linear polysaccharide derived from chitin, the second most abundant natural biopolymer, biocompatible and non-immunogenic. More importantly, chitosan presents a high elastic modulus and a good porosity, necessary parameters for cell migration and integration. It presents similarities with glycosaminoglycans, components of the ECM. Its abundance in nature combined with its low production cost makes it a very interesting polymer for use in bioengineering [59,60,61]. However, the low-cell adherent characteristic needs to be improved with other materials, like collagen, to increase the tissue integration and mechanical stability [62]. Alginate-hydrogels, based on an anionic polysaccharide, are described to reinforce scar thickness and to improve cardiac function after MI [63,64]. Nevertheless, their major limitation is the lack of integration within cardiac tissue and the relative stiffness conferred by alginate, an undesirable property for the heart compared to other hydrogels. It was recently reported that thermosensitive poly(ethylene glycol) (PEG)-hydrogels and alginate gels, with an increased elasticity, reduced infarct size 4 weeks after injection compared to the injection of a saline solution [65]. In this regard, natural and synthetic biomaterials may be combined to enhance their therapeutic potential for cardiac repair. The choice of the type of biomaterial to be used in cardiac regeneration studies remains to be further explored; nevertheless, its therapeutic usefulness is now recognized as undeniable.

It is also imperative to determine when the therapeutic strategy should be administered. To this end, a recent study [66] envisaged the injection of chitosan hydrogel at 3 different times: just after MI, 3 days after MI and 2 weeks after MI, which correspond to the beginning of the necrotic, fibrotic and chronic remodeling phases, respectively. They evaluated the histological and functional outcome 10-weeks post-MI hydrogel injection. They thus demonstrated that, although at all injection times the hydrogel had a positive effect on LV function and wall thickness, the group of rats injected 3 days after MI had better functional results compared to the other groups. A better local vascularization and fewer inflammatory markers were observed at this time point compared to the group receiving the hydrogel right after MI. The time of administration of the therapy is therefore crucial. In addition, it is reported that, after MI, one of the most important problems encountered is the non-conductivity of the scar tissue, which is formed in the border of the ischemic tissue and contributes to ventricular dysfunction. To correct this, the injection of a chitosan-based hydrogel in in vivo models has shown propagation and synchronization of contraction leading to restoration of LV function by restoring conduction between cardiomyocytes [67]. Biological evaluation of these chitosan hydrogels revealed that they could be injected into the epicardial surface of the heart but showed only partial degradation. In addition, mononuclear cell infiltration was demonstrated. It is important that the future work on chitosan hydrogels focuses on accelerating resorption kinetics and on promoting macrophagic infiltration as well as their polarization towards an anti-inflammatory M2 phenotype, promoting tissue reconstruction.

Numerous studies have also been conducted associating injectable scaffolds with cells and therapeutic molecules for cardiac repair after MI. The objective is to obtain a prolonged therapeutic strategy to provide anchorage support for cells, which is essential for their survival but also act as a reservoir of GF that will repair the damage of the ischemic cardiac tissue.

### 2.2. Scaffolds as Cell or GF Carriers

It has been shown that seeding human MSCs in a fibrin cell carrier allows their effective delivery to a targeted area and improves regional mechanical function after infarction [68]. Among the synthetic and biodegradable polymers used in cell therapy, poly(lactide-co-glycolide) acid (PLGA) is an FDA- and European Medecines Agency-approved biomaterial currently used to produce cells microcarriers. These PLGA microcarriers when functionalized with a biomimetic surface provide a 3-D anchorage support for the cells stimulating their survival [43,69]. Using these microcarriers, it has been shown in a chronic MI rat model that the transported human adipocyte-derived stem cells (hADSCs) showed increased engraftment compared to hADSCs transplanted alone [70]. Similarly, it was recently shown that the combination of poly-D-lysine (PDL) and collagen-coated PLGA microcarriers with cardiomyocytes derived from human iPSCs allows detection of cells up to two months after grafting at the border of an MI [71]. This clearly shows that the use of this type of cell carrier considerably improves cell survival but also allows a therapeutic effect since the studies show an improvement in cardiac function mediated by a paracrine effect of the cells. An injectable hydrogel transporting hADSCs also showed that it may not only provide a cell-supporting action; it appears that its use in an in vivo model increases the secretion of many proangiogenic factors, including VEGF, HGF or FGF-2, by the transplanted hADSCs [72]. In this way, their cardiovascular repair potential through their proangiogenic or anti-inflammatory action is improved.

In recent years, several teams have associated scaffolds with pro-angiogenic factors, such as VEGF and PDGF-BB [73] or bFGF [74], to favor the formation of a vascular network, necessary in cardiac regeneration following MI. Despite the beneficial effects observed, the effects were sometimes transient and may be attributable to the low stability and short half-life of these released cytokines. Thus, it is necessary to combine GF with injectable biomaterials that allow site-specific targeting and regenerative action by increasing the half-life of the GF or by obtaining a sustained release to achieve a prolonged therapeutic effect after MI. Studies have demonstrated good retention and progressive release of GF, such as HGF, VEGF or bFGF, due to the properties of the scaffolds. A positive effect on cardiac regeneration through the pro-angiogenic and arteriogenic actions improving ventricular function and preventing fibrosis and cardiac hypertrophy in different MI models has been reported in these studies [75,76,77,78]. Equally, albumin-alginate microcapsules allow progressive release of GF such as FGF, HGF and IGF during 28 days. These microcapsules incorporated in a collagen hydrogel induced cardiac regeneration by favoring angiogenesis as well as stimulation, recruitment and proliferation of endogenous cardiac stem cells following an acute MI [79]. Interestingly, it has also been shown that a synthetic, biocompatible and injectable nano-silicate hydrogel can deliver the secretome of hADSCs [80]. This method allows prolonged delivery of the secretome over time at the injection site that is essential for optimal therapeutic efficacy by effectively promoting key therapeutic mechanisms such as angiogenesis, scar surface reduction and cardioprotection.

Other factors can also promote cardiac regeneration. Indeed, the administration of a hydrogel containing factor 6-bromoindirubin-3-oxime (BIO), in combination with IGF has been shown to protect against apoptosis and post-MI LV dilatation. This hybrid hydrogel, where gelatin nanoparticles allow the co-release of BIO and IGF, not only improves cardiomyocyte proliferation and the subsequent cardiac function after MI due to the prolonged release of these factors but also provides mechanical properties specific to hydrogels [81]. Furthermore, it has been shown that the encapsulation of IL-10 in natural microgels using an emulsion technique improves cardiac function after MI due to anti-inflammatory properties [82]. In addition, the use of a thermosensitive PLGA-PEG-hydrogel as a vehicle for colchicine, an anti-inflammatory agent released in a sustained manner during 8 days, showed that a single intramyocardial injection alleviated cardiac inflammation, inhibited myocardial apoptosis and fibrosis, and improved cardiac function and structure after MI [83]. Finally, in another approach, knowing that MI creates a hypoxic microenvironment, Fan et al. (2018) were interested in the prolonged release for 4 weeks of oxygen encapsulated in PLGA microspheres included in a hydrogel. The study showed that the prolonged release of oxygen resulted in increased cell survival and cardiac regeneration [84]. Through these different studies, we highlight the importance of combining approaches and not restricting the strategy to a single therapeutic actor. Thus, it could be of interest to study the effects of a multiple combination based on the association of scaffolds allowing the support of cells and the enrichment of the microenvironment with GF.

### 2.3. Scaffolds and GF Combined to Promote Cell Engraftment and Repair

Despite the beneficial effect of using a cell carrier as an anchorage for cells, their use as a vehicle alone remains limited and could be complemented by a role as a GF reservoir allowing the joint action of cells, biomaterials and therapeutic biological factors. Indeed, a cell and GF delivery carrier may have dual functions. First, both the cells and the delivered GF may exert a direct action on the microenvironment to protect the endogenous cells in the lesion site. Secondly, the GF delivered by the carrier may also act on the transplanted cells themselves to favor their integration and thus their function. Studies have demonstrated that the intramyocardial injection of the combination of adipocyte-derived stem cells (ADSC) with fibronectin (FN)-coated microcarriers delivering IGF and HGF injected in a 2-week-old MI model allowed an increase in vascularization of the infarcted area after 2 weeks of treatment. Not only did the microcarriers increase the survival and engraftment of the transported cells having a regenerative action but also the concomitant release of two GF promoted cardiac regeneration through their proangiogenic and cell survival effects [70]. In this respect, an in vitro study showed that the release of IGF and HGF by these microcarriers stimulated the expression of markers of cardiac differentiation such as GATA4, Nkx2.5, cTnI or CX43 [85]. Similarly, Hahn et al. (2008) showed that a pretreatment with IGF and HGF had a cytoprotective effect and improved the therapeutic efficacy of transplanted MSCs in myocardial repair due to the priming of MSCs by the GF [86]. In addition, the association of ADSCs with FN-coated and VEGF-releasing microcarriers resulted in improved cardiac function and better tissue integration of ADSC into ischemic heart tissue after 21 days of treatment in mice compared to ADSCs alone or to the conditioned media of ADSCs [87].

For the moment, it remains important to remember that the effects obtained in cardiac regeneration with the use of cell therapy, with or without scaffolds, remain very limited and seem to be quite equivalent to the long-term effects obtained with the secretome secreted by these same cells [88,89,90,91,92]. Indeed, GFs are not the only actor exerting an effective paracrine action in the secretome, Extracellular vesicles (EVs) also mediate a large number of effects and are able to have a beneficial therapeutic action after MI. Cell carriers are largely used for cells [48,93], but they could also convey EVs, without the side effects observed with cells partially due to the induced immune response.

## 3. Extracellular Vesicles

**Figure 3:**
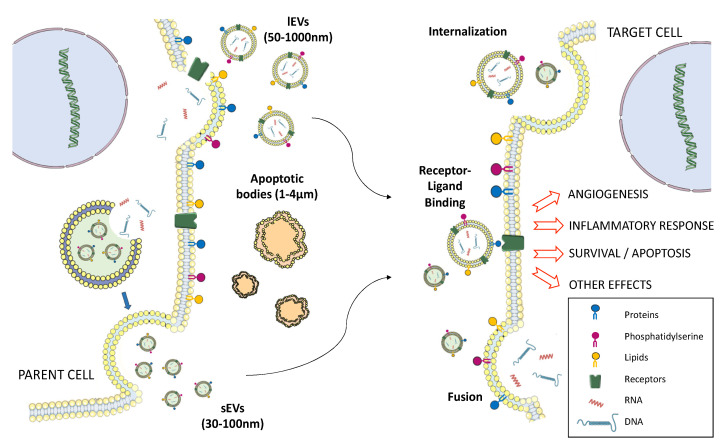
Different types of interactions between extracellular vesicles (EVs) and target cells.

Extracellular vesicles (EVs) are released by cells during both physiological and pathological phenomena [94,95,96,97]. Three types of vesicles involved in the intercellular communication and released into all body fluids can be globally distinguished [98,99]. First is apoptotic bodies (size between 1 and 4 µm), which originate from cells undergoing apoptosis that fragment into these bodies containing the information present in the cell cytoplasm. Then, there are the microvesicles (size between 50 and 1000 nm), known as large EVs (lEVs) as defined in the nomenclature. They result from budding of the cytoplasmic membrane caused by an input of calcium into the cell and from reorganization of the cytoskeleton causing the exposure of phosphatidylserine outside the membrane [100]. Finally, there are exosomes (size between 30 and 100 nm), also known as small EVs (sEVs). They differ from lEVs by their biogenesis pathway. Indeed, they originate from endosomal multivesicular bodies and are released from the cells after fusion of these compartments with the plasma membrane [98]. Their isolation is based on density and size parameters and will not be described in this review (for details, see [101,102]). EVs interact in different ways with recipient cells (Figure 3), leading to the modification of the function of cells or the induction of several signaling pathways. It occurs following the interaction between ligands carried by EVs and receptors present in the target cells and/or the internalization of EVs into target cells by membrane fusion, endocytosis (clathrin-dependent or -independent by lipid draft and caveolin) or phagocytosis [103].

It has been shown that, in a mouse model of MI, sEVs and lEVs are released and carry markers of cardiomyocytes and endothelial cells [104]. This production of endogenous cardiac EVs, described as proinflammatory, locally produced after MI, are taken up by monocytes infiltrating the ischemic heart and subsequently increase their proinflammatory response. This endogenous EV release aims to initiate beneficial endogenous repair triggered by the MI but is often insufficient for complete myocardial repair.

### 3.1. Myocardial Repair with Cell-Derived EVs vs. with Cells Alone

The study of EVs effects after MI has generated a great deal of interest [105,106]. Even if all cells present in the myocardium are able to generate EVs such as cardiomyocytes, endothelial cells, platelets and leukocytes [107], interest has focused on EVs derived from cells that have already shown therapeutic interest in clinical studies for cardiac regeneration (Table 2).

A comparative study of the effect of MSC-derived sEVs and MSCs showed that EV therapy was more effective in limiting cardiac fibrosis, in inflammation and in improving cardiac performance [108]. Indeed, numerous studies have demonstrated that MSC-derived EVs are effective in the treatment of MI and ischemic reperfusion injury, reporting a reduction in the size of the infarction and an improvement in cardiac function [109,110,111,112]. Mechanisms allowing these benefits are multifactorial due to a joint action of antiapoptotic, anti-inflammatory, antioxidant and pro-survival effects of EVs [113]. Despite the benefits observed during treatment with EVs, it is important to note that the use of cell injection-based therapy remains advantageous when the cells are able to integrate the tissue and to replace the lost cells or when they survive for a prolonged period of time. Indeed, in this way, the beneficial effects last longer with longer-term secretion of soluble factors and EVs.

The study initiated by Dr. Ménasché’s team on embryonic stem cells (ESC) has shown that EVs from hESC possess the same beneficial therapeutic effect as the cells themselves [114]. In parallel, a study conducted on in vivo effects of mouse ESC-derived EVs revealed an improvement in cardiac functionality and cardiomyocyte survival and an induction of neovascularization in a mouse model after 8 weeks post-MI [115]. However, few studies are conducted with these cells due to their origin, but it reinforces the hypothesis that the beneficial effects obtained in cardiac regeneration could be attributed to EVs. As indicated above, iPSCs possess a strong regenerative potential [116]. The 10 proteomic analysis of iPSC-derived EVs revealed molecules that promote cardiac, endothelial and smooth muscle cell proliferation and that protect against oxidative damage [117]. Moreover, iPSC-derived EVs do not lead to teratoma formations like iPSC while carrying similar therapeutic agents beneficial to cardiac regeneration. In view of this therapeutic potential, a comparative study was conducted 48 h after I/R with iPSCs and iPSC-derived EVs used both in in vitro and in vivo mouse models to determine the cardiac repair capacities. The iPSC-derived EVs induced in vitro angiogenic, migratory and antiapoptotic properties in cardiac endothelial cells and induced superior in vivo infarct repair compared with iPSCs 35 days after I/R [117]. It might be interesting to establish whether the superior therapeutic benefit observed with EVs is related to higher efficacy and rapidity of action or to the low cell survival of iPSCs after 35 days, which does not provide the therapeutic effect necessary to obtain the same cardiac regeneration.

Another alternative was based on the use of cardiac progenitor cells (CPCs) for cell therapy. Some studies have shown that CPC-derived EVs had cardioprotective and proangiogenic effects in pig [118] and murine models [119,120]. They showed that these effects could be partially attributed to the miRNA content carried by EVs [119]. Moreover, one study showed that CPC-derived EVs could potentially repair injured cardiac tissue mainly through endogenous cardiac stem cell homing and activation in the lesion site [120]. Also, it has been shown that intravenous injection of CPC-derived sEVs displayed cardioprotective effects via the overexpression of miRNA. Indeed, these miRNA decrease the cardiotoxicity induced by the combination of doxorubicin/trastuzumab used in cancer therapy [121].

In addition, given the massive influx of immune cells following cardiomyocyte necrosis, the therapeutic effect of EVs released by immune cells was studied. More specifically, one study investigated the EVs derived from dendritic cells, described as contributing to immune responses. Dendritic cell-derived sEVs have been shown to improve cardiac function after MI via CD4^+^ T lymphocyte activation [122], which plays a key role in improving myocardial wound healing after MI [123]. Among the cells that are strongly involved at the site of vessel obstruction in an MI are platelets, which aggregate into a compact structure within the artery when the atheroma plaque ruptures. In this regard, it has been shown that platelet-derived EVs can induce angiogenesis in vitro by the activation of different pathways, such as Src, PI-3K and ERK signaling in endothelial cells, which lead to the secretion of VEGF, bFGF and PDGF [124].

Other approaches have considered the beneficial action of the protection against oxidative stress for cardiac repair. They studied the use of EVs carrying heat shock protein (HSP), allowing good protein folding. Indeed, the relationship of HSP and cardioprotection is well-established [125]. It has also been shown that plasma sEVs isolated from healthy humans and adult rats were powerfully cardioprotective in all tested models of heart ischemia-reperfusion injury, since these vehicles expressed cardioprotective HSP70. The mechanism of plasma EV-mediated cardioprotection involves HSP70-dependent activation of Toll-like receptor 4 (TLR) followed by activation of cardioprotective HSP27 in cardiomyocytes [126].

All of these studies have reported that the release of EVs leads to, at least, the same or better therapeutic benefit in the treatment of MI as the cells themselves [127,128]. These findings support the idea that EVs play a key role in the therapeutic effects observed in terms of functional restoration of the myocardium or cell differentiation.

### 3.2. Use of Modified EVs as Therapeutic Agents to Improve Native Beneficial Effects of EVs

**Figure 4:**
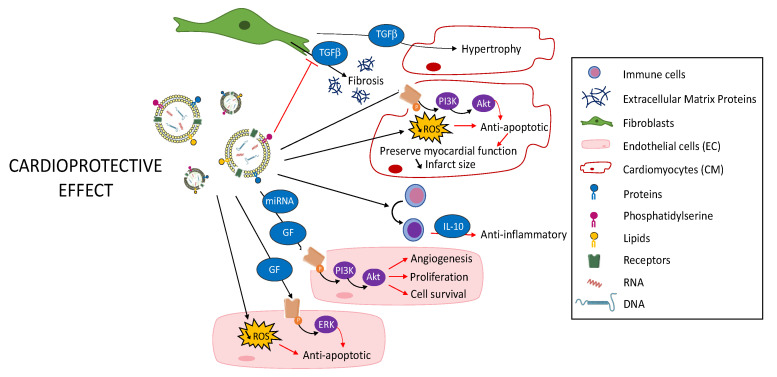
Cardioprotective effect of modified EVs.

In addition to the use of stem cell-derived EVs described above, one possibility to enhance their therapeutic effect is to modify the content of the EVs by the integration or overexpression of new molecules, RNA fragments or proteins to limit microenvironment damages induced by ischemia (Figure 4). To obtain these EVs, a first approach consists in directly modifying the released EVs by loading them with molecules or proteins of interest, giving them an additional therapeutic character. A second approach consists in premodifying the parent cells in order to obtain EVs with a modified content.

Lymphocyte-derived EVs have been engineered to modify their cargo with the aim of improving their therapeutic potential. Indeed, the EVs were passively loaded with curcumin, an anti-inflammatory molecule [129]. It was suggested that this approach could be an efficient way to administer anti-inflammatory molecules to the lesion site to regulate inflammation, which is the initial phase generated by MI. In addition, the microenvironment resulting from ischemia is particularly harmful, and the loading of molecules to protect EVs from possible degradation can be suitable. In this regard, curcumin loading increased the stability and delivery of EVs.

Among the most widely described modifications of the content of sEVs is miRNA loading to mediate therapeutic effects. In particular, it has been shown that sEVs released by hypoxia-exposed endothelial cells were enriched with miRNA-126 and miRNA-210. Both miRNAs possess proangiogenic properties, which are necessary to induce the formation of a new vascular network to limit MI-related cell death. They increased cardiac progenitor cell resistance to hypoxic stress through activation of PI3K/Akt and other prosurvival pathways [130,131]. These same miRNAs are found in MSC-derived EVs and may decrease MI [132]. It has also been shown that overexpression of miRNA-21 in parent cells, an antiapoptotic miRNA, effectively restored cardiac function after MI by improving wound healing and myogenesis through the miRNA-21-carrying EVs [132,133]. The use of miRNA-carrying EVs for cardiac regeneration will not be further discussed in this review as it has been covered elsewhere (for more details, see [96,132,134,135]).

Another approach to optimize the beneficial therapeutic effect of EVs by premodifying the parent cells used a model of transfection of cardiosphere-derived cells by short noncoding Y RNA. The generated EVs enriched with these RNAs reduced inflammation caused by ischemia. When administered at the ischemic heart, the Y RNA-enriched EVs reduced the number of macrophages and modified their polarization leading to an increased expression of anti-inflammatory genes such as *IL4RA*, *VEGFA* or *TGFB1*. In addition, secretion of the anti-inflammatory IL-10 by these macrophages promoted cardiomyocyte protection from oxidative stress and reduced infarct size [136]. EVs may also be modified by overexpressing proteins involved in developmental signaling pathways in parent cells. To illustrate this point, lymphocytes constitute an important source of EVs that may exert pro- or antiangiogenic effects depending on the stimuli involved in their production. When lymphocytes undergo activation before apoptosis, they release proangiogenic lEVs and their use in in vitro and in vivo mouse models showed that they were able to stimulate functional vessel formation [137,138]. In addition, lEVs expressing the morphogen sonic hedgehog, a proangiogenic factor, were able to decrease ROS production and their in vivo injection in mice was also able to improve endothelial function by increasing NO release and to reverse endothelial dysfunction after myocardial I/R [139]. Furthermore, Mackie and colleagues engineered CD34^+^ stem cells able to release sEVs overexpressing sonic hedgehog. Injection of these modified sEVs to the border zone of murine hearts after MI preserved cardiac function through the reduction of infarct size [140]. Other studies have demonstrated that genetically MSC-derived sEVs overexpressing GATA4, a key regulator of cell surviving pathways and cardiac genes, reduced infarct size after an acute MI by transfer of antiapoptotic miRNA [141].

The ability to deliver EVs having an efficient and specific therapeutic activity for cardiac tissue remains a major challenge. Indeed, it is essential to minimize potential off-target effects on other organs before considering any potential switch to the clinic. For this purpose, a novel targeting system to improve sEV uptake by cardiomyocytes in vitro and then in vivo consists of modifying the parent cells to express a fusion protein. This protein is engineered to contain a cardiac-targeting peptide (CTP), known to allow addressing to cardiomyocytes, and Lamp2b, known to be involved in the production of sEVs, leading to sEVs carrying CTP-Lamp2b on their surface. Thus, a study has shown a 16% uptake improvement of CTP-Lamp2b-sEVs by cardiomyocytes both in vitro and in vivo after intravenous injection [142]. A similar strategy showed that targeted sEVs, also expressing a fusion protein between another peptide and Lamp2b, resulted in an increased uptake by cardiomyocytes in vitro as well as in vivo following intramyocardial administration. They decreased cardiomyocyte apoptosis and showed a higher cardiac retention after injection compared to nontargeted sEVs [143]. This interesting strategy needs to be confirmed through a long-term biodistribution study. By the same approach, another cardiac homing peptide, CHP, has been identified to specifically target ischemic myocardium [144,145]. The presence of CHP on the surface of cardiosphere-derived sEVs allows increased retention of sEVs in the ischemic heart in a rat MI model [146]. It also allows a beneficial therapeutic effect, superior to that of non-CHP sEVs, through the reduction of both MI size and fibrosis and of angiogenesis [146]. Equivalent therapeutic results were obtained with the use of murine MSC-derived sEVs and an enriched membrane protein (Lamp2b) fused with ischemic myocardium-targeting peptide [147]. This suggests that, in addition to the therapeutic action exerted by the sEVs, the mode of administration has a major role in the biodistribution of the therapeutic strategy and showed a better therapeutic efficacy against cardiac dysfunction induced by MI.

### 3.3. Why Are EVs Not Yet Transferred to the Clinic?

Despite many successful preclinical studies, to our knowledge, there are still no data showing cell-derived EV effects in MI in patients. However, several phase I/II clinical trials are ongoing to evaluate the application of EVs in cancer patients [148,149], suggesting that the risk–benefit balance is still too weak to rapidly consider clinical trials using EVs on cardiovascular pathologies. Indeed, current treatments, although limited in terms of cardiac regeneration, are sufficient to limit and reduce patient mortality. It is therefore important to continue preclinical studies in order to envisage a translation to the clinic. Indeed, EVs present similar or even superior therapeutic properties to the parental cells, which have largely demonstrated their immunomodulatory and cardiac regeneration properties in preclinical and clinical studies. However, ethical concerns, particularly with the use of ESCs, and safety concerns, with the possible formation of teratoma with the use of iPSCs or precursor cells, still remain. Moreover, despite the ease of isolation of adult stem cells, their expansion limits and their restrictive plasticity do not make them prime candidates for clinical use. EVs are an acellular therapy presenting multiple modes of action and are weakly immunogenic, thus constituting an alternative to cell and to pharmacological treatments [150].

As with all therapy tools considered for clinical transfer, some points still need to be improved for EVs to rapidly reach the clinic. One of the main causes of failure in clinical trials is the lack of standardization and rigor in cell recovery procedures, and the same problem is also present for EVs. Indeed, despite the implementation of standardized isolation procedures, there are still too many variations delaying the use of EVs in the clinic, including variations in purification protocols involving a potential heterogeneity of EV populations. Nevertheless, their easy access and their high therapeutic potential due to their ability to cross physiological barriers make them ideal candidates for future clinical use. Despite numerous studies on EVs in recent years, some parameters, such as the subpopulation of EVs, which mediate therapeutic effects, and the mode of administration still need to be established in order to limit off-target effects on other organs, triggering toxicity or undesirable tumors. One of the options considered to limit biodistribution in the body, making the use of EVs unsafe, would be to consider their association with scaffolds, already known and used as a support for cell therapy.

## 4. EVs Association with Scaffolds as a Future Direction

Numerous studies have shown similar therapeutic potential between cells and EVs combining anti-inflammatory and antiapoptotic actions with proangiogenic and cardioprotective actions. In light of the studies carried out, cell-free therapy is very attractive because of the low immunogenicity and robust effect on ischemic cells and more advantages than cell therapy. Among the listed off-target effects of EVs, it appears essential to find a way to retain them in the intracardial injection site before considering their use in the clinic. This could be envisaged by associating them with scaffolds used as carriers for which the aim would be to confine them and, if possible, to gradually release them thereafter for a sustained effect. Different sterilization approaches will be required before a transfer to clinical use of carriers can be considered. Among the various techniques already known, particularly for the sterilization of microcarriers, are cold γ-irradiation [151,152], radiosterilization [153,154], the use of ethylene oxide [155,156] or obtaining these carriers by formulation under sterile conditions. It will be imperative to validate the use of these techniques in order to verify the quality and integrity of the carriers, with or without proteins, after these steps before considering their association with EVs. This association has been tested in cardiac regeneration, which has shown that sEVs, released by cardiomyocytes-derived from iPSC, delivered by a patch hydrogel decreased cardiomyocyte apoptosis and arrhythmic burden 24 h after implantation. In addition, the therapeutic action goes on until 4 weeks post MI by a reduction of the infarct size and a decrease in cell hypertrophy [157]. This delayed action may be attributed to the prolonged delivery of sEVs by the hydrogel. Other studies have shown that sEVs-derived endothelial progenitor cells (EPC), associated with an intracardially injected hydrogel, improved angiogenesis and promoted myocardial haemodynamic after MI [158,159]. These studies also demonstrated that the therapeutic action was carried by the EVs as the hydrogel alone did not improve myocardial function and the hydrogel containing the EPCs had the same beneficial effect on haemodynamic function as the sEV hydrogel [158]. In parallel, another study showed that MSC-derived EVs administered by an alginate hydrogel decreased cardiac cell apoptosis and promoted macrophage polarization quickly after MI and long-term cardiac function. In this study, it has been shown that EVs injected in a hydrogel are highly sustained in the heart and scarcely present in liver, lungs and spleen as observed for EVs injected alone [160]. These studies support the hypothesis that the progressive release of EVs allowed for a therapeutic effect while limiting undesirable biodistribution in the body. All these elements confirm that different scaffolds can be considered in order to develop the full therapeutic potential of EVs in a more efficient way than using cells. It could be particularly attractive to study the combined effect of EVs associated with the scaffold, releasing GF as a combinatorial therapeutic approach for cardiac repair. However, further studies, particularly in vivo studies, still need to be carried out to confirm the safety of this approach.

## Figures and Tables

**Table 1:** Benefits and limits of different cell types used in clinical trials for regenerative medicine after MI. * Clinical trial with the use of a cardiac patch.

Cell Type	Benefits	Limits	Clinical Trial
Skeletal myoblasts	Abundant, contractile properties, withstand ischemic insult	Committed to skeletal muscle lineage, high mortality after injection	MAGIC
Bone Marrow Mononuclear Cells (BMMC), Mesenchymal Stem Cells (MSC), Hematopoietic Stem Cells (HSC)	Easy acquisition, rapid proliferation, multipotency, low immunogenicity, immune-privileged, potential for allogenic use	Heterogeneous cell population, lack of standardized study methodologies, lack of long-term follow-up to determine if benefits will last	BOOST BALANCE BONAMI (NCT00200707) REGENERATE-AMI (NCT00765453) MI3-Trial SWISS-AMI (NCT00355186) REPAIR-AMI SCAMI (NCT00669227) TIME (NCT00684021) LateTIME (NCT00684021) BAMI (NCT01569178) REGENT (NCT00316381) COMPARE-AMI PROCHYMAL (NCT00114452) PROCHYMAL II (NCT00877903) MyStromaCell (NCT01394432) Precise Trial (NCT00426868) RELIEF (NCT01652209) ESTIMATION (NCT01394432) SEESUPIHD (NCT02666391) C-CURE (NCT00810238) CHART (NCT01768702) WJ-MSC-AMI (NCT01291329)
Cardiac Stem Cells (CSC)	Higher differentiation potency into cardiac lineages	Invasive isolation procedure, high expansion cost, low cell availability, older/autologous donors means lower quality cells	CADUCEUS (NCT00893360) CAREMI NCT02439398) ALLSTAR NCT01458405) SCIPIO (NCT00474461)
Embryonic Stem Cells (ESC)	Pluripotent	Ethical, political and availability issues	ESCORT (NCT02057900) *

**Table 2:** Therapeutic effects in preclinical studies with EVs after MI or ischemia-reperfusion (I/R) in different animal models.

EV Source	Assay Model	Function and Mechanism	Mechanism	References
MSC	IM rat, intramyocardial injection	Preserve myocardial function	Endogenous miRNA	[108]
		Reduce fibrosis		
		Inhibit fibroblast transformation		
		Promote cardiomyocyte proliferation		
Human MSC	I/R mouse, intravenous injection	Increase systolic function	Activation of survival pathways	[109]
		Decrease infarct size	Decrease neutrophils and macrophage infiltration	
		Decrease inflammation		
MSC	MI rat, intramyocardial injection	Promote angiogenesis	miRNA-150, HIF, SHH, PDGFR carrying by EVs	[110]
		Preserve cardiac performance		
MSC	I/R mouse, intravenous injection	Cardioprotective effect	Paracrine effect of EVs, undefined	[111]
MSC	MI rat, intramyocardial injection	Improve cardiac function	Undefined	[112]
		Decrease fibrosis		
		Promote angiogenesis		
iPSC	I/R mouse, intramyocardial injection	Attenuate LV dysfunction and hypertrophy	Protection against oxidative damage	[117]
		Reduction of myocyte apoptosis		
		Enhance angiogenesis		
CPCs (murine cardiosphere- derived cells)	I/R mouse, intramyocardial injection	Decrease cardiomyocyte apoptosis	Possibly related to miRNA144 et miRNA451 content in EVs and secretion of soluble factors	[120]
Human CPCs	IM rat, intramyocardial injection	Improve cardiac function	miRNA carrying by EVs	[119]
		Reduced cardiomyocyte apoptosis		
		Enhance angiogenesis		
CPCs (Human cardiosphere-derived cells)	I/R pig, intracoronary injection	Decrease infarct size, collagen content	Undefined	[118]
		Decrease cardiomyocyte hypertrophy		
		Increase vessel density		
Embryonic stem cells (ESC)	AMI mouse, intramyocardial injection	Enhance cardiac function	Recruitment of endogenous CPCs (c-kit^+^ cells)	[115]
		Reduce fibrosis		
		Enhance neovascularization		
		Enhance cardiomyocyte survival		
Human ESC-derived CPCs	IM mouse, intramyocardial injection	Increase cardiac function	Undefined	[114]
		Decrease fibrosis and cardiomyocyte hypertrophy		
		Increase capillary/cardiomyocyte ratio		
Dendritic cells	MI mouse, intravenous injection	Improve cardiac function	Activation LT CD4^+^ (endocrine mechanism)	[122,123]
Platelet	MI rat, intramyocardial injection	Increase capillary formation	Action of cytokines (bFGF, PDGF, VEGF)	[124]
Rat and human plasma	I/R rat, intravenous injection	Decrease infarct size	Activation of HSP70/TLR4 protective pathway	[126]
